# Autophagic Heterogeneity in Gastric Adenocarcinoma

**DOI:** 10.3389/fonc.2021.555614

**Published:** 2021-03-30

**Authors:** Ju-Yoon Yoon, Christine Brezden-Masley, Catherine J. Streutker

**Affiliations:** ^1^ Department of Laboratory Medicine and Pathobiology, University of Toronto, Toronto, ON, Canada; ^2^ Department of Pathology, St. Michael’s Hospital, Toronto, ON, Canada; ^3^ Department of Hematology/Oncology, St. Michael’s Hospital, Toronto, ON, Canada

**Keywords:** autophagy, gastric/GEJ adenocarcinoma, autophagy (macroautophagy), chaperone-mediated autophagy, biomarker

## Abstract

**Background and Aim:**

Gastric/gastroesophageal junction (GEJ) adenocarcinoma is a heterogeneous disease, with various etiologies and with tumors encompassing a spectrum of histologic and molecular subtypes. “Autophagy” includes two related but distinct homeostatic processes that promote cell survival under adverse conditions, namely macro- and chaperone-mediated autophagy. There is increasing evidence of the roles autophagy may play in tumorigenesis.

**Methods:**

Autophagic pathways were examined in the context of the heterogeneity intrinsic to gastric/GEJ adenocarcinoma, utilizing immunohistochemistry targeting specific proteins within the pathways (p62, LAMP2A, LC3B). We examined whole sections of normal and dysplastic gastric mucosa, as well as a tissue microarray of adenocarcinomas.

**Results:**

Dysplastic gastric epithelium was marked by frequent nuclear p62 and aberrant LAMP2A expression compared to normal. Examining the pattern of LC3B/cytoplasmic p62 immuno-reactivity in gastric adenocarcinoma demonstrated a predominant pattern of LC3B^High^/p62^High^ staining (56/86, 65.1%), which has been previously associated with active, but impaired macroautophagy. There were no statistically significant associations seen between LC3B/cytoplasmic p62 staining patterns with tumor grade, histotype, or approximated TCGA molecular subtype. LAMP2A and nuclear p62 and staining patterns were also heterogeneous across the cohort, but with no statistically significant associations seen. The prognostic significance of the three proteins was limited, however high nuclear p62 levels were associated with worse overall survival (log-rank *p*-value = 0.0396).

**Conclusion:**

Our data demonstrate the dynamic nature of autophagic proteins in the gastric epithelium, and we expand the biological heterogeneity observed in gastric/GEJ adenocarcinoma to include autophagy.

## Introduction

Metabolic homeostasis is maintained by several pathways, including autophagy. Autophagy is a cellular process which allows the cell to remove damaged components and to “recycle” normal cytoplasmic constituents. This is an important protective mechanism in epithelial cell homeostasis and cell survival; for example, inhibiting autophagy in an animal model of ethanol exposure results in increased gastric epithelial cell death and ulceration (1). The term “autophagy” includes several different biological processes including macroautophagy, microautophagy, and chaperone-mediated autophagy (CMA), although “autophagy” is often used as a synonym for macroautophagy. Macroautophagy involves sequestration of different cytosolic constituents and digestion of the content by autophago-lysosomes (2). Macroautophagy involves both the conversion of LC3B to LC3B-II as well as consumptive degradation of various contents in and on the autophago-lysosome, including p62. The role of p62 (also known as sequestosome 1/SQSTM1) is multifaceted in cancer biology and includes a role in regulating the NF-κB signaling pathway (3). In comparison to macroautophagy, CMA involves substrates binding to LAMP2A, an isoform form of LAMP2, a lysosomal membrane protein (4, 5). LAMP2A serves as a receptor for the chaperone-substrate complexes, and LAMP2A mediates translocation of the substrate into the lysosomal lumen, where the substrate undergoes degradation (5).

The clinical significance of autophagy in cancer patients is difficult to study, partially related to technical challenges in studying a biologically dynamic process in cancer tissues. Numerous studies have examined the prognostic significance of a few autophagy-related proteins, including Beclin-1, LC3, and p62, with heavy bias towards macroautophagy [reviewed in (6)]. However, as most studies have focused on single proteins, associating their expression levels to autophagy is challenging. As well, gastric cancer is considered to be a markedly heterogeneous malignancy, encompassing multiple etiologies which vary across populations/ethnicities, including infections (including *Helicobacter* and Epstein-Barr Virus), chronic inflammatory states (ex. autoimmune gastritis), and hereditary conditions (ex. Lynch and Hereditary diffuse gastric cancer syndromes) (7). Gastric adenocarcinoma also demonstrates a spectrum of histologic/architectural changes, with the majority being either intestinal type or diffuse type cancers. Focusing on gastric/GEJ adenocarcinoma molecular studies, The Cancer Genome Atlas (TCGA) study has subdivided these malignancies into four molecular subtypes, namely EBV-related, microsatellite instability (MSI), chromosomal instability (CIN), and genome stable (GS) (8, 9).

In this study, we aimed to examine the significance of autophagy in gastric cancer, while addressing the heterogeneity both in autophagy (macroautophagy *vs.* CMA) and in gastric cancer (histotype and molecular subtypes). We examined the levels of three autophagy-related proteins, MAP1LC3B (LC3B), p62, and LAMP2A, by IHC in the normal-to-carcinoma spectrum, and we describe their heterogeneous expression patterns in our cohort of gastric/GEJ adenocarcinoma.

## Materials and Methods

### Study Cohort and Immunohistochemistry

This study was performed in conjunction with our institution’s research ethics board (SMH REB 10-280). The cohort comprises gastric adenocarcinoma cases treated at the St. Michael’s Hospital (Toronto, Ontario, Canada), treated with either gastrectomy or endoscopic mucosal resection (EMR), between the period 2001 to 2011. This cohort has been described in our previous reports (10). A tissue microarray (TMA) was constructed as described previously (10, 11), consisting of two 0.6 mm cores per each tumor, with several corresponding normal cores. Histology subtypes were obtained from the pathology reports associated with each case, and diffuse histology was interpreted as per the Lauren classification. A series of cases of dysplastic gastric mucosa (low- and high-grade dysplasia, n = 21) were examined as whole slides and compared to specimens with normal mucosa or intestinal metaplasia (n = 19).

Immunohistochemistry (IHC) was performed using the Novus anti-LC3B antibody (NB100-2220) that recognizes both LC3-I and LC3-II isoforms, at the 1:200 dilution, with citrate buffer antigen retrieval. Anti-p62 IHC was performed using an antibody from BD Biosciences (#610832) at the 1:100 dilution, and the anti-LAMP2A IHC was performed using an antibody from AbCam (ab125068) at the 1:400 dilution. For both IHCs, antigen retrieval was performed using a Tris/EDTA buffer. The Roche Ventana BenchMark ULTRA system was used for all IHCs.

In scoring LC3B and p62 IHC, we adopted a previously published scoring scheme that correlated the extent of cancer cells showing dot-like staining with autophagic activity (12–14). However, because we observed dot-like staining in the majority of cancer cells in gastric adenocarcinoma, IHC staining was scored in a qualitative method that combined the intensity (absent to strong) and extent (absent, patchy, diffuse), from 0 (absent/no staining) to 3+ (strong and diffuse). Representative cores are pictured in **Supplementary Figure 3**. Cases with more diffuse staining, i.e. 2+/3+ staining for LC3B and cytoplasmic p62 were classified as “high,” reflecting the frequent dot-like staining pattern observed in our cohort. A similar scoring method was adopted for LAMP2A. For nuclear p62, IHC was scored from 0 (none), 1+ (focal), 2+ (patchy), 3+ (diffuse nuclear staining); while the staining intensity did vary from case to case, the most notable differences were in the extent of nuclear staining.

### Approximation of the Molecular Subtypes

We had previously described our method for approximation of the molecular subtype (11). Briefly, we employed a subtyping algorithm based on the TCGA algorithm, a series of dichotomizing steps. We first identified the EBV-CIMP cases, identified by EBER positivity. The MSI subtypes were next identified through immunohistochemistry (IHC) for mismatch repair (MMR) pathway proteins, MLH1, PMS2, MSH2, and MSH6. Among the remaining MMR intact, EBER-negative cases, the remainder was subdivided into CIN and GS subtypes. CIN and GS subtypes were divided based on the histotypes (diffuse *vs.* intestinal/mixed).

### Statistics

Survival analyses were performed using the Kaplan-Meier method. Comparisons of categorical variables (including IHC staining patterns) between multiple groups were performed using ANOVA/Chi-square tests. All statistical tests were performed using JMP (SAS version 13/14).

## Results

### Autophagy Marker Expression Across the Normal-Carcinoma Spectrum in the Stomach

**Figure 1:**
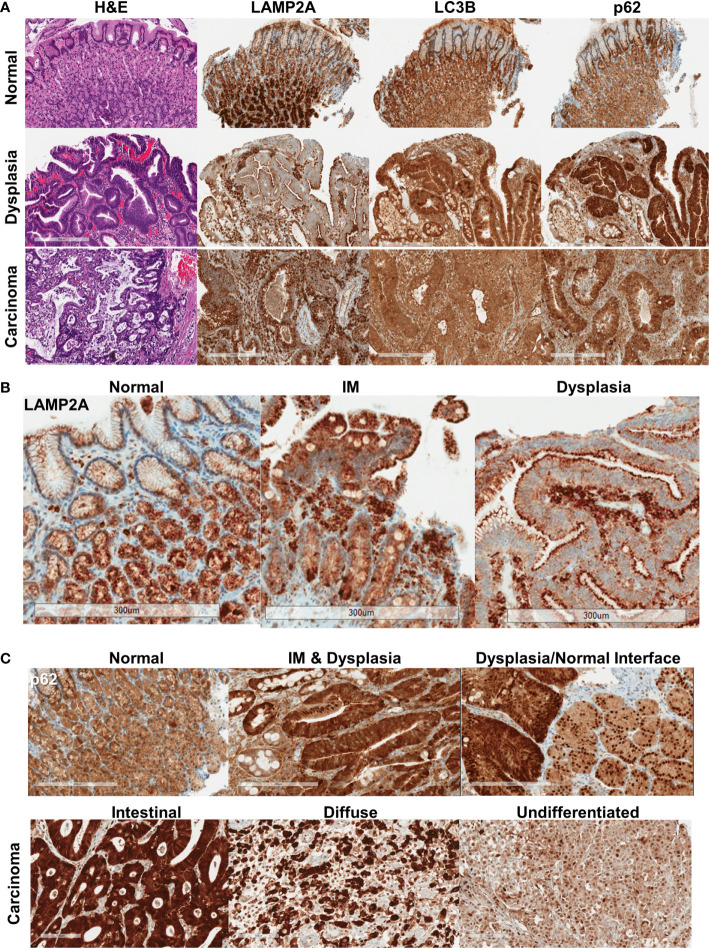
**(A)** Protein expression pattern of three autophagy markers in non-dysplastic, dysplastic, and carcinomatous gastric epithelium. **(B)** LAMP2A expression patterns seen in normal, intestinal metaplasia (IM) and dysplastic surface mucosa. **(C)** p62 expression patterns, with the bottom panel showing the different expression patterns in different histotypes of carcinoma. Bar = 300 μm.

Normal gastric mucosa and mucosa with intestinal metaplasia showed relatively homogeneous, moderate levels of p62 and LC3B immunostaining (n = 19, **Figure 1A**, **Supplementary Figure 1**). p62 expression in the normal mucosa was limited to the cytoplasm. LAMP2A expression was strongest in the base of the gastric glands, with much weaker expression near the surface (**Figures 1A, B**, **Supplementary Figure 1**). In dysplastic mucosa (both low- and high-grade), a combination of aberrant LAMP2A expression pattern and frequent nuclear p62 expression was consistently observed (n = 21, **Figures 1B, C**, **Supplementary Figures 1**, **2**). The surface-base gradient seen in LAMP2A expression in the normal mucosa was aberrant in both intestinal metaplasia and dysplasia, with some of the cases showing numerous, dark puncta in the apical aspects, comparable to the pattern seen in normal base (**Figure 1B**). p62 in dysplastic mucosa showed generally stronger cytoplasmic expression, accompanied by frequent nuclear p62 staining (**Figure 1C**, **Supplementary Figures 1**, **2**). Interestingly, in some of the areas where we captured the dysplastic/non-dysplastic mucosal interface, increased nuclear p62 could be observed in the bordering normal mucosa (**Figure 1C**). Dysplastic mucosa also showed stronger cytoplasmic, granular LC3B staining (**Figure 1A**, **Supplementary Figure 1**).

In invasive adenocarcinoma, variable staining patterns were observed (representative cores for each IHC score are shown in **Supplementary Figure 3**). LC3B was often granular and cytoplasmic, with a dot-like pattern being observed in tumors with stronger staining (**Figure 1A**, **Supplementary Figure 2**). LAMP2A, as in dysplasia, was seen with apical accentuation in some well-differentiated tumors (**Supplementary Figure 3**). Punctate and diffuse cytoplasmic LAMP2A was seen in other tumors. Examining the relationship between the proteins, stronger nuclear p62 was seen more frequently with stronger cytoplasmic p62 (*p* = 0.0097) (**Supplementary Figure 4**). LC3B tended to be weaker when there was stronger nuclear p62, and stronger LAMP2A was seen with stronger nuclear p62, but these associations did not reach statistical significance. Weaker LAMP2A was generally seen with stronger (2+/3+) LC3B staining (*p =* 0.0183).

### Autophagic Heterogeneity in Gastric/GEJ Adenocarcinoma

**Figure 2:**
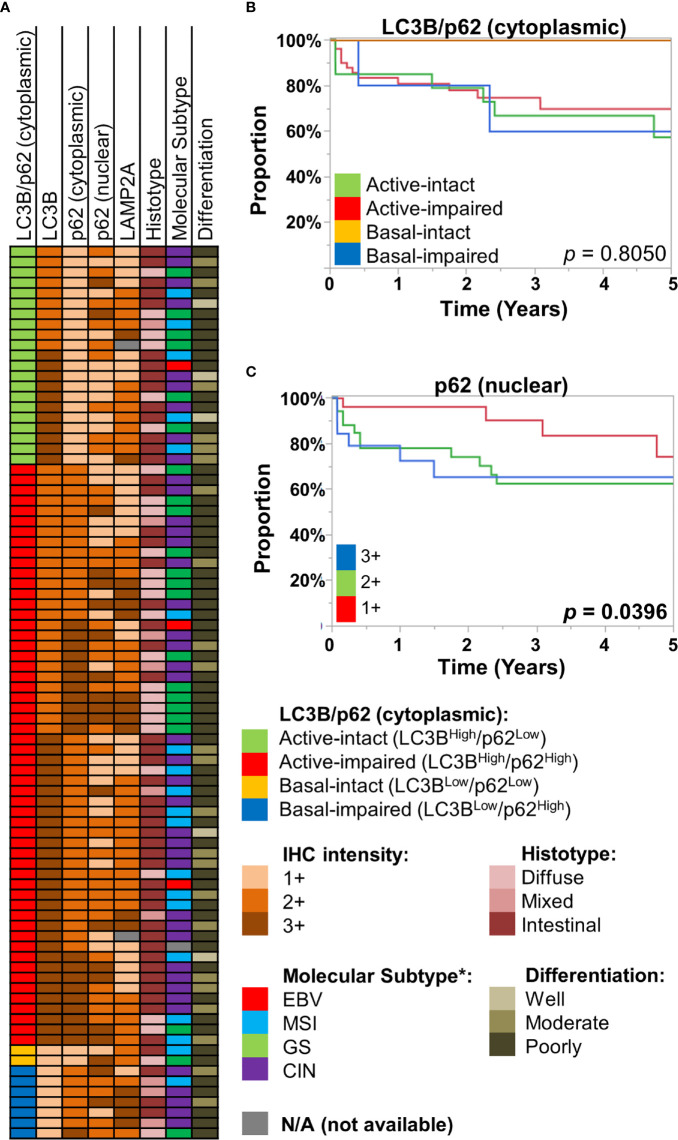
**(A)** Case information for the 86 gastric/gastro-esophageal junction (GEJ) adenocarcinoma cases, displaying their LC3B/p62 (cytoplasmic) staining patterns, along with immunohistochemical (IHC) intensity for LC3B, p62 (cytoplasmic, nuclear), and LAMP2A for each case. Also displayed are their respective histotype, approximated TCGA molecular subtype (see *Methods*), and tumor differentiation. **(B, C)** Kaplan-Meier curves, showing overall survival of the St. Michael’s gastric/GEJ adenocarcinoma cohort, divided by **(B)** macroautophagic subtypes (LC3B/cytoplasmic p62), and **(C)** nuclear p62 intensity.

We employed an IHC scoring scheme previously described by Schläfli et al. to our cohort of gastric/GEJ adenocarcinoma (n = 86), examining the combined IHC patterns of LC3B and (cytoplasmic) p62 staining patterns, in which the active-impaired pattern (LC3B^High^/p62^High^) was the most common combination (56/86, 65.1%). This was followed by active-intact (LC3B^High^/p62^Low^, 21/86, 24.4%), basal-impaired (LC3B^Low^/p62^High^, 7/86, 8.1%), and basal-intact (LC3B^Low^/p62^Low^, 2/86, 2.3%). There were no statistically significant associations between LC3B/p62 staining patterns with tumor grade (differentiation), histotype (intestinal, diffuse, or mixed), or approximated TCGA molecular subtypes (**Figure 2A**, **Supplementary Figure 5**). Similarly, no statistically significant associations were observed between LAMP2A and nuclear p62 staining with tumor grade, histotype, and approximated TCGA molecular subtypes (**Figure 2A**, **Supplementary Figure 5**).

Previous studies had examined the prognostic value of autophagy-related proteins in gastric cancer. For example, reduced Beclin-1, which regulates autophagy, has been associated with worse prognosis in gastric cancer by several studies (15–18). However, autophagy being a dynamic process, simply equating Beclin-1 with autophagic activity is difficult. Examining LC3B, cytoplasmic p62, and LAMP2A as individual markers, there was no statistically significant difference in overall survival (OS) (**Supplementary Figure 6**). The LC3B/cytoplasmic p62 combination was also of little prognostic significance; most of the survival curves were largely overlapping, with exception of the basal-intact macroautophagy group, with no deaths in this small group (0/2). In contrast, nuclear p62 was significantly prognostic, where 2+ or 3+ staining was associated with worse OS (log-rank *p* = 0.0396) (**Figure 2C**).

Taken together, our results suggest autophagy, both macroautophagy and CMA, are dynamic processes in the stomach, across the normal-to-carcinoma spectrum. IHC patterns of the three proteins are variable across the different pathological attributes, including approximated molecular subtypes. Prognostic significance of the IHC patterns were limited, but nuclear p62 expression was associated with worse survival.

## Discussion

Assessing autophagy is difficult in pathology specimens. Various cellular assays for autophagy rely on live tissues, often examining for the autophago-lysosome fusion and associated events (19). This can be done using various dyes and/or fluorescent-tagged proteins (ex. LAMP2A, p62). In cell lysates, immunoblotting for the formation of LC3B-II isoform is another popular technique. While we employed IHC to assess autophagy, not all of the immuno-reactivity may be related to macroautophagy *per se*. LAMP2A also plays key roles in CMA (4), and the LAMP2A staining pattern was quite distinct from that of LC3B and p62 (**Figure 1A**), suggesting that some of the LAMP2A expression may be related to CMA. In the normal epithelium, LAMP2A was strikingly stronger in the glands at the base of the mucosa; this orientation becomes lost in dysplastic epithelium, with frequent apical accentuation. Compensatory upregulation of CMA has been described in the setting of defective macroautophagy (4), and, accordingly, strong LAMP2A was seen more frequently with impaired autophagy (cytoplasmic p62^High^) in our adenocarcinoma cohort. Also, about 1/3 of gastric carcinomas have been shown to exhibit immunoreactivity for lysozyme components, supported by ultrastructural (electron microscopic) findings, which has been described as abortive expression of Paneth cell differentiation (20, 21). More recent reports have described “secretory autophagy,” where Paneth cells can utilize an alternative, macroautophagy-based system, involving the formation of LC3-decorated, autophagosome-like structures, followed by release of the contents into the intestinal lumen (22). In this regard, the LAMP2A staining pattern seen in IM, dysplastic mucosa and well-differentiated adenocarcinoma is interesting; we more frequently observed apical accentuation (**Figure 1B**), raising the possibility that secretory autophagy, potentially through at least partial differentiation toward Paneth cells in intestinalized mucosa, may be contributing to the LAMP2A pattern observed. While our tissue sample preparation method (formalin-fixed, paraffin-embedded) precludes electron microscopy, future studies would benefit from correlations with ultrastructural analyses.

Interpreting the pattern of LC3B and cytoplasmic p62 staining in combination, we observed that active-impaired macroautophagy (LC3B^High^/cytoplasmic p62^High^) was the most frequent phenotype in our cohort. While increased degree of impaired autophagy (cytoplasmic p62^High^) was seen more frequently with moderately and poorly differentiated tumors, the distribution of the different macroautophagy subtypes was relatively even across the different pathological attributes, including molecular subtypes. Accordingly, no significant survival difference was observed by the different macroautophagy patterns; while the patients with basal-intact phenotype were notable for good prognosis, this subgroup only contained two patients for the survival analysis (**Figure 2B**). This is in contrast to colorectal carcinoma, where active-impaired macroautophagy less frequently observed, and it was reported to be associated with worse survival (12). Distinguishing our negative finding from a type II error would likely require a much larger cohort.

In the cytosol, p62 co-localizes with LC3 as part of the autophagosome, and p62 is continuously cleared in the setting of intact autophagy (23, 24). Nucleo-cytoplasmic shuttling of p62/SQSTM1 has been previously described, regulation of which, among other mechanisms, may be controlled by phosphorylating of p62 at the CDK1 site (25). In this study, stronger (2+/3+) nuclear p62 was associated with worse survival (**Figure 2C**). These results conflict with those reported by Mohamed et al., who observed no significant differences in survival in their cohort of 61 gastric carcinoma patients (26). Several explanations are possible, including relatively small cohort sizes in both studies and different proportion of clinically advanced cases in our cohorts (23/61 *vs.* 69/86 advanced cases in our cohort). In the dysplastic gastric mucosa, our data suggests that macroautophagy may often be impaired, leading to accumulation of both cytoplasmic and nuclear p62. Interestingly, accumulation of p62 in autophagy-defective cells has been shown to result in defective recruitment of DNA repair proteins (27). The combination of impaired macroautophagy with the accumulation of nuclear p62 may thus be playing key roles gastric carcinoma pathogenesis and suggest a possible mechanism by which field effect and subsequent cancer spread may be occurring. As well, considering the well-established role of p62 in different signaling pathways, including the NF-κB pathway (3), we suspect nuclear p62 accumulation may have multifaceted impact on the cell survival-death balance.

While limited by our small cohorts, our results nonetheless demonstrate the dynamic expression pattern and relationship between LAMP2A, LC3B, and p62, the combination of which point to a possible relationship of tumor development with macroautophagy and CMA. The LC3B/cytoplasmic p62 combination suggests that autophagy is often impaired in gastric/GEJ adenocarcinoma, which may be contributing to nuclear p62 accumulation. While the mechanism behind the significance of nuclear p62 is unclear, its prognostic significance suggests a role for p62 in disease progression.

## Data Availability Statement

The original contributions presented in the study are included in the article/**Supplementary Material**. Further inquiries can be directed to the corresponding author.

## Ethics Statement

The studies involving human participants were reviewed and approved by St. Michael’s Hospital research ethics board. The ethics committee waived the requirement of written informed consent for participation.

## Author Contributions

J-YY contributed to the design of the work, data interpretation, and manuscript writing. CB-M contributed to survival data development and manuscript writing. CS contributed to the design of the work, data interpretation, and manuscript writing. All authors contributed to the article and approved the submitted version.

## Conflict of Interest

The authors declare that the research was conducted in the absence of any commercial or financial relationships that could be construed as a potential conflict of interest.

## Abbreviations

CIN, chromosomal instability; CMA, chaperone-mediated autophagy; EBV, Epstein-Barr Virus; EMR, endoscopic mucosal resection; GEJ, gastro-esophageal junction; GS, genomic stability; IHC, immunohistochemistry, immunohistochemical; MMR, mismatch repair; MSI, microsatellite instability; TCGA, The Cancer Genome Atlas; TMA, tissue microarray.
