# Supplementary material for: Autophagic Heterogeneity in Gastric Adenocarcinoma
Source: Front Oncol. 2021 Mar 30;11:555614. doi: 10.3389/fonc.2021.555614 (PMC8042205; doi:10.3389/fonc.2021.555614)
Supplement: Supplementary file 1 [file Presentation_1.pptx]

## Slide 1
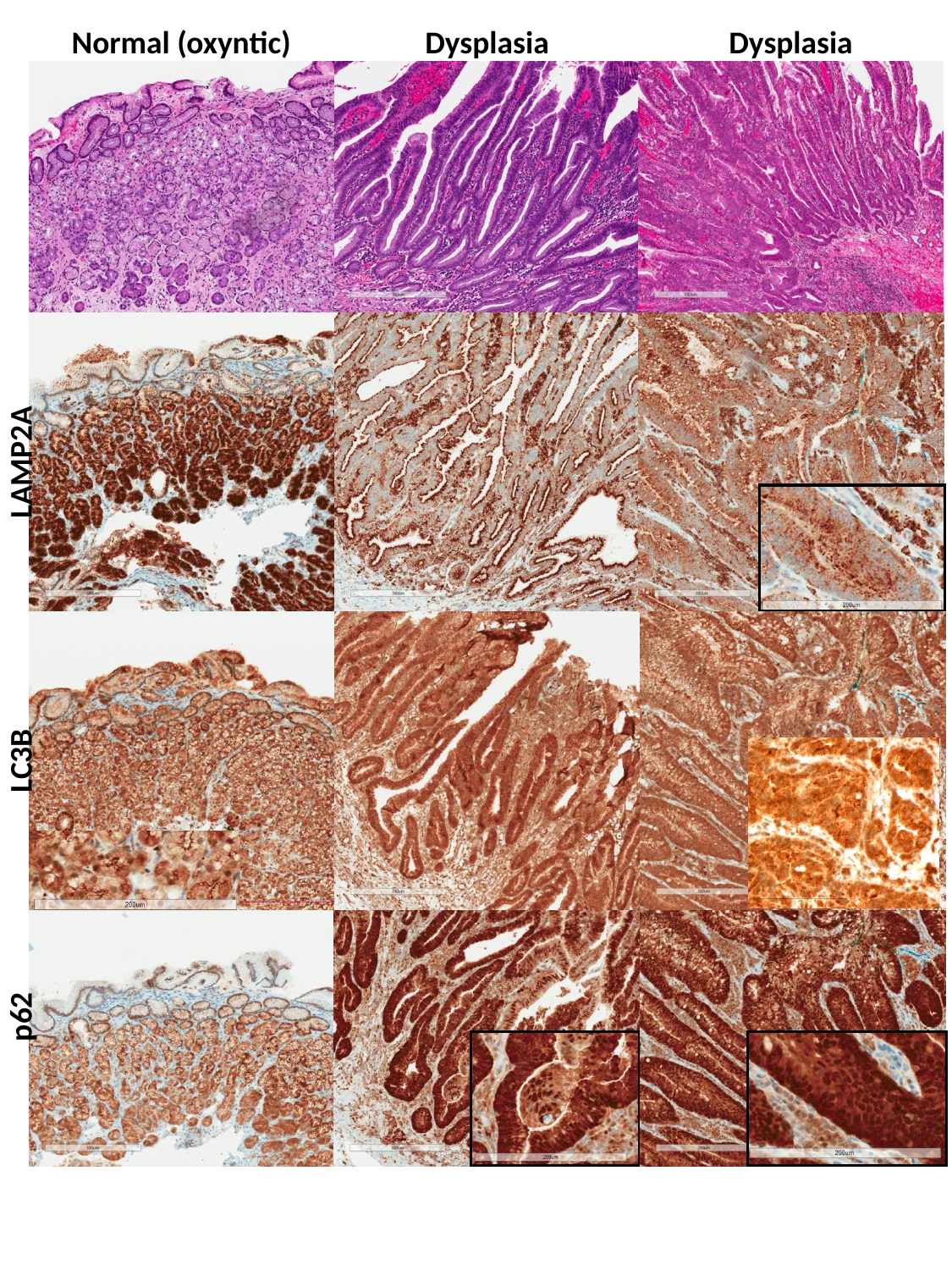

Normal (oxyntic)
Dysplasia
Dysplasia
LAMP2A
LC3B
p62

## Slide 2
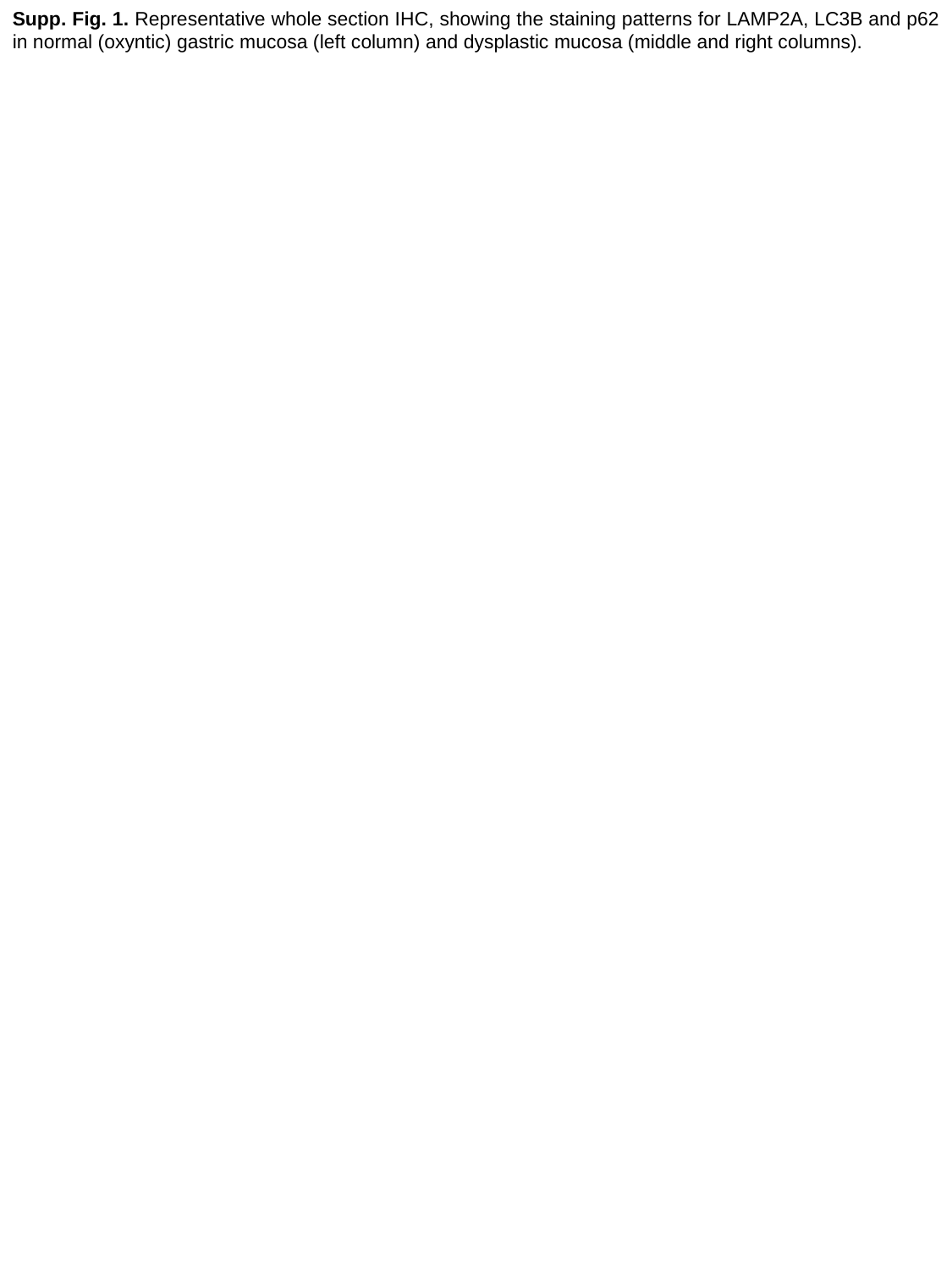

Supp. Fig. 1. Representative whole section IHC, showing the staining patterns for LAMP2A, LC3B and p62 in normal (oxyntic) gastric mucosa (left column) and dysplastic mucosa (middle and right columns).

## Slide 3
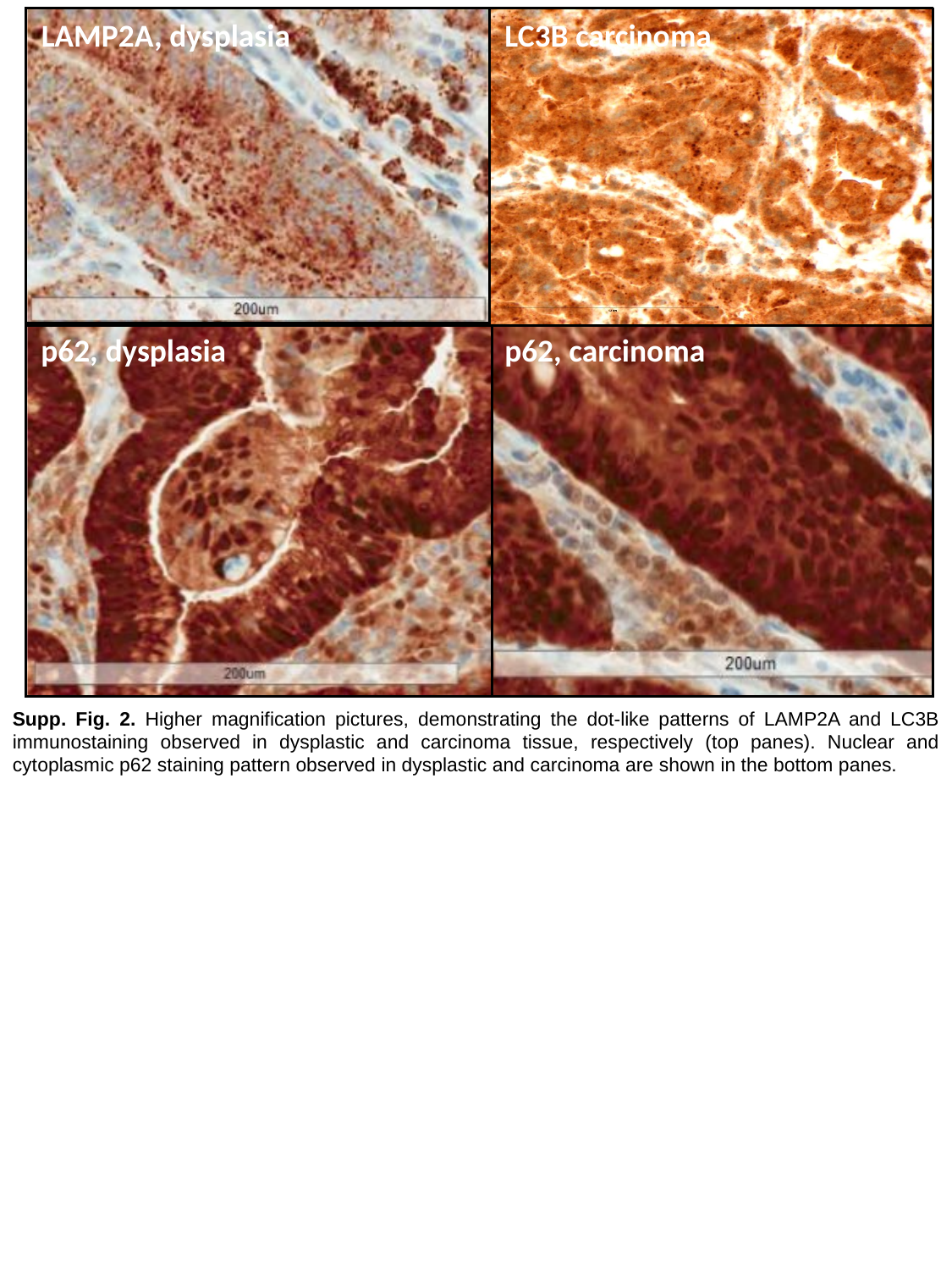

LC3B carcinoma
LAMP2A, dysplasia
p62, carcinoma
p62, dysplasia
Supp. Fig. 2. Higher magnification pictures, demonstrating the dot-like patterns of LAMP2A and LC3B immunostaining observed in dysplastic and carcinoma tissue, respectively (top panes). Nuclear and cytoplasmic p62 staining pattern observed in dysplastic and carcinoma are shown in the bottom panes.

## Slide 4
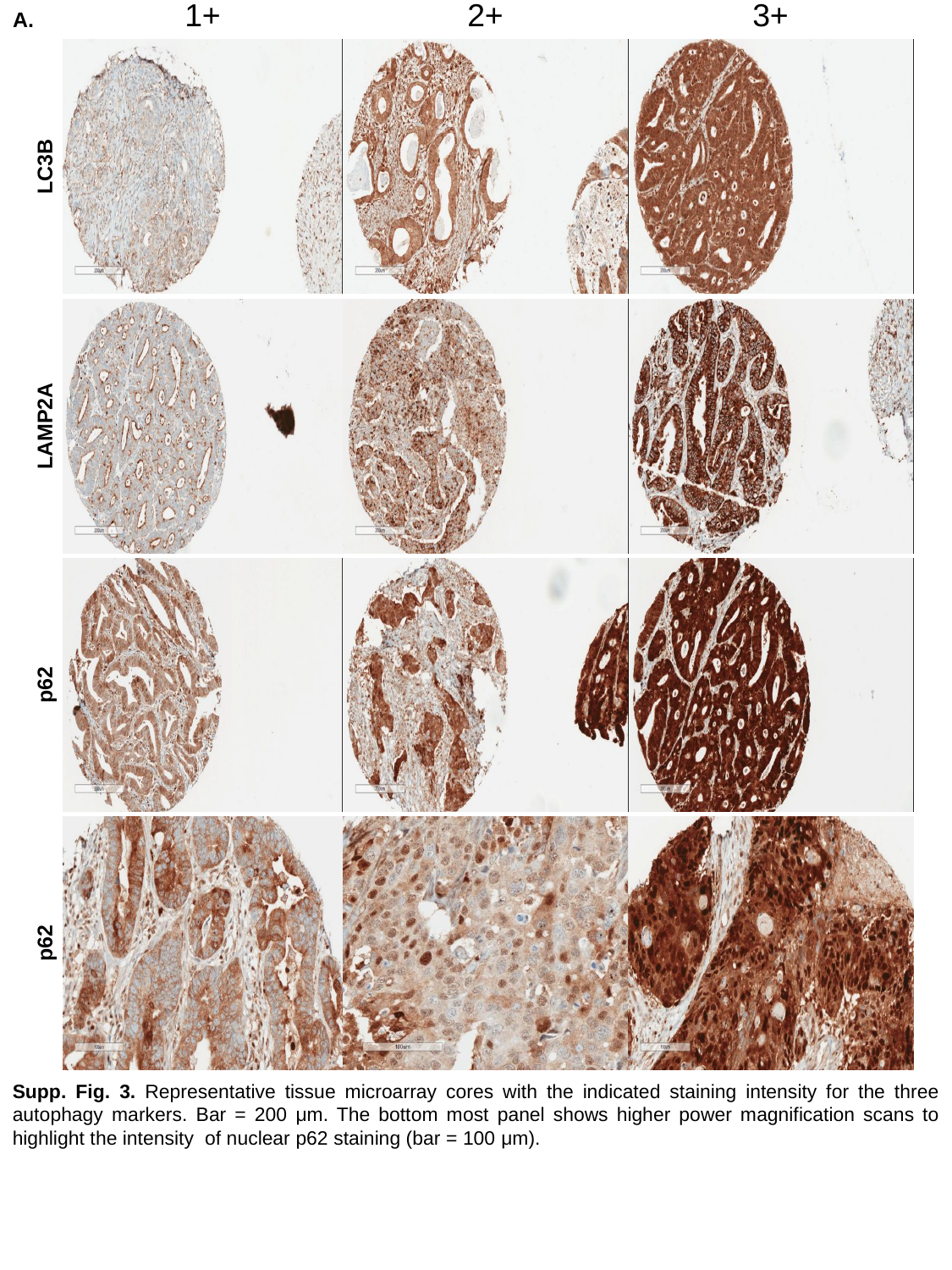

A.
1+
2+
3+
LC3B
LAMP2A
p62
p62
Supp. Fig. 3. Representative tissue microarray cores with the indicated staining intensity for the three autophagy markers. Bar = 200 μm. The bottom most panel shows higher power magnification scans to highlight the intensity of nuclear p62 staining (bar = 100 μm).

## Slide 5
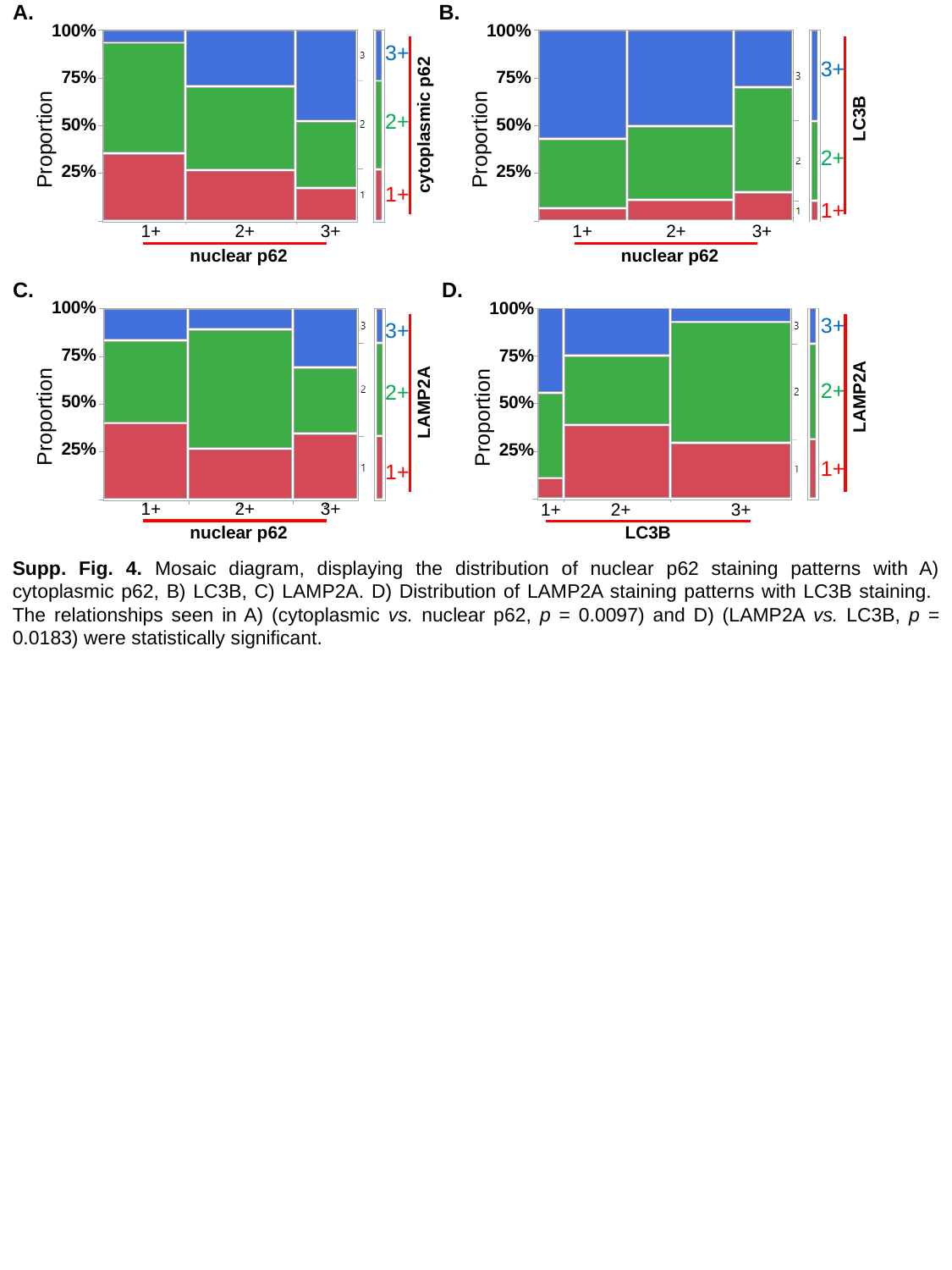

A.
B.
| 100% |
| --- |
| 75% |
| 50% |
| 25% |
| 100% |
| --- |
| 75% |
| 50% |
| 25% |
3+
3+
LC3B
2+
cytoplasmic p62
Proportion
Proportion
2+
1+
1+
1+
2+
3+
1+
2+
3+
nuclear p62
nuclear p62
C.
D.
| 100% |
| --- |
| 75% |
| 50% |
| 25% |
| 100% |
| --- |
| 75% |
| 50% |
| 25% |
3+
3+
2+
2+
LAMP2A
LAMP2A
Proportion
Proportion
1+
1+
1+
2+
3+
1+
2+
3+
LC3B
nuclear p62
Supp. Fig. 4. Mosaic diagram, displaying the distribution of nuclear p62 staining patterns with A) cytoplasmic p62, B) LC3B, C) LAMP2A. D) Distribution of LAMP2A staining patterns with LC3B staining. The relationships seen in A) (cytoplasmic vs. nuclear p62, p = 0.0097) and D) (LAMP2A vs. LC3B, p = 0.0183) were statistically significant.

## Slide 6
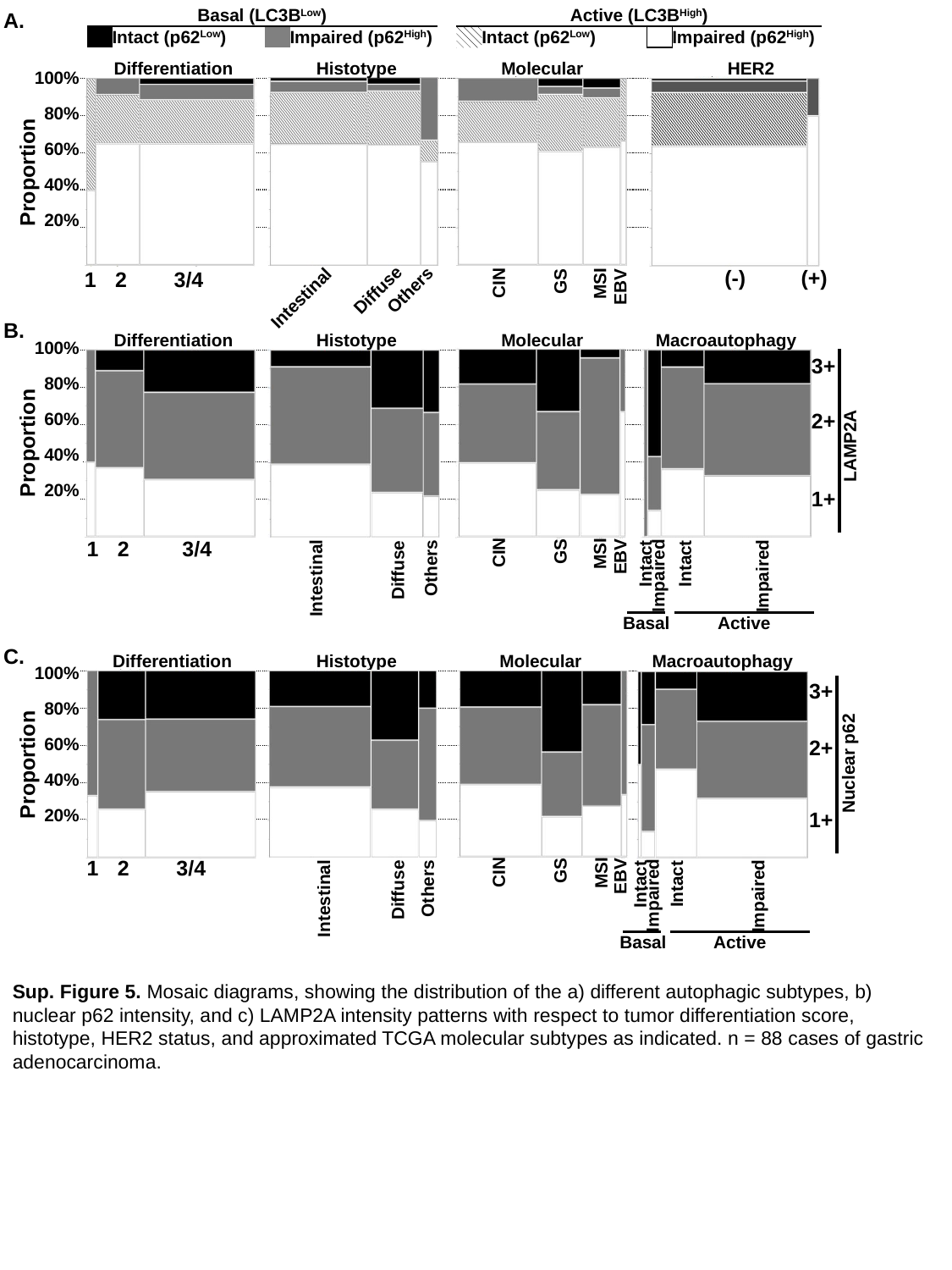

A.
| Basal (LC3BLow) | | | | | Active (LC3BHigh) | | | |
| --- | --- | --- | --- | --- | --- | --- | --- | --- |
| | Intact (p62Low) | | Impaired (p62High) | | | Intact (p62Low) | | Impaired (p62High) |
Differentiation
Histotype
Molecular
HER2
| 100% |
| --- |
| 80% |
| 60% |
| 40% |
| 20% |
Proportion
(-)
(+)
1
2
3/4
GS
CIN
MSI
EBV
Diffuse
Others
Intestinal
B.
Macroautophagy
Differentiation
Histotype
Molecular
| 100% |
| --- |
| 80% |
| 60% |
| 40% |
| 20% |
3+
2+
Proportion
LAMP2A
1+
1
2
3/4
GS
CIN
MSI
EBV
Intact
Intact
Others
Diffuse
Impaired
Impaired
Intestinal
Basal
Active
C.
Differentiation
Histotype
Molecular
Macroautophagy
| 100% |
| --- |
| 80% |
| 60% |
| 40% |
| 20% |
3+
2+
Proportion
Nuclear p62
1+
1
2
3/4
GS
CIN
MSI
EBV
Intact
Intact
Others
Diffuse
Impaired
Impaired
Intestinal
Basal
Active
Sup. Figure 5. Mosaic diagrams, showing the distribution of the a) different autophagic subtypes, b) nuclear p62 intensity, and c) LAMP2A intensity patterns with respect to tumor differentiation score, histotype, HER2 status, and approximated TCGA molecular subtypes as indicated. n = 88 cases of gastric adenocarcinoma.

## Slide 7
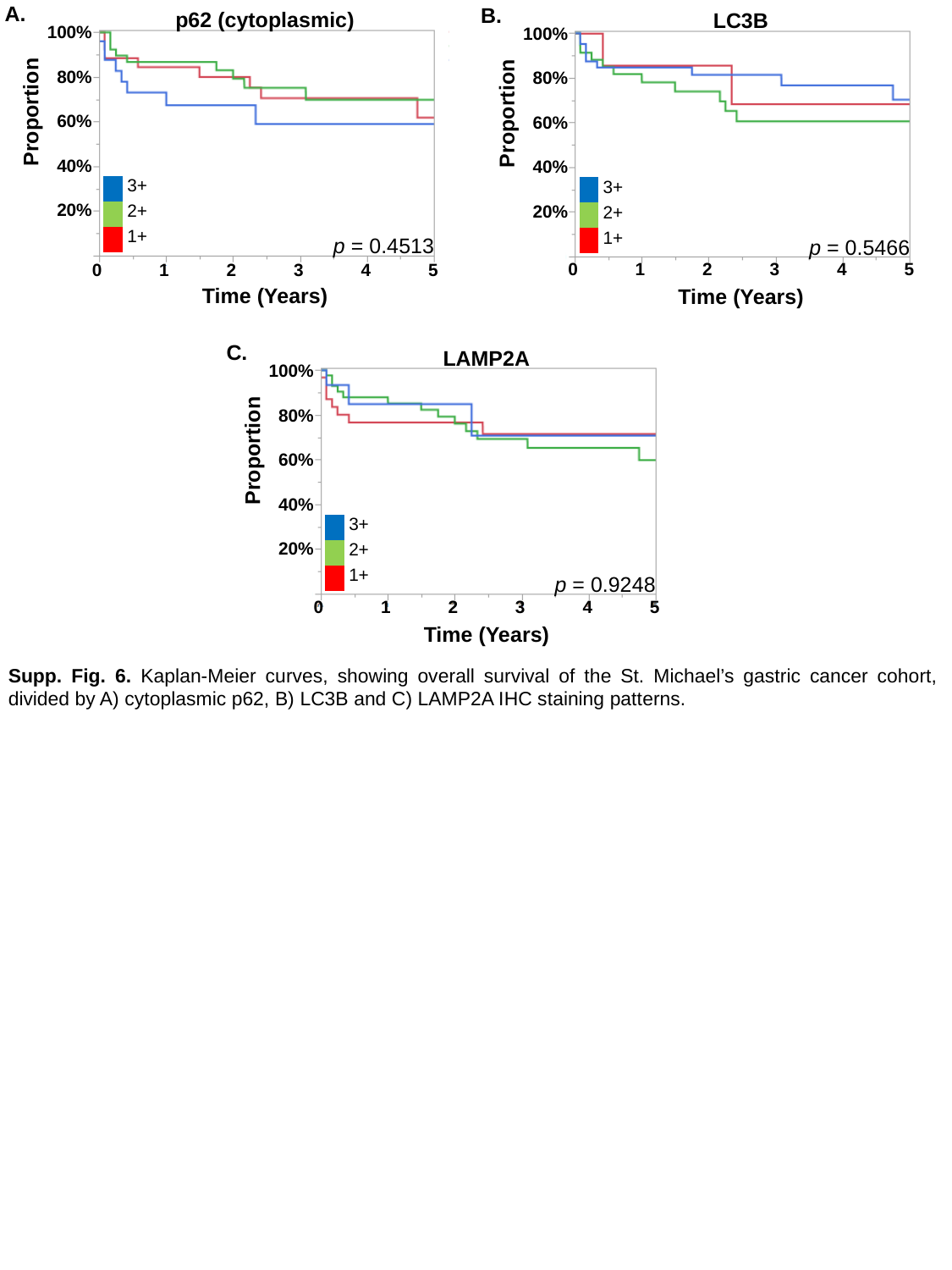

A.
B.
p62 (cytoplasmic)
LC3B
| 100% |
| --- |
| 80% |
| 60% |
| 40% |
| 20% |
| 100% |
| --- |
| 80% |
| 60% |
| 40% |
| 20% |
Proportion
Proportion
| | 3+ |
| --- | --- |
| | 2+ |
| | 1+ |
| | 3+ |
| --- | --- |
| | 2+ |
| | 1+ |
p = 0.4513
p = 0.5466
| 0 | 1 | 2 | 3 | 4 | 5 |
| --- | --- | --- | --- | --- | --- |
| 0 | 1 | 2 | 3 | 4 | 5 |
| --- | --- | --- | --- | --- | --- |
Time (Years)
Time (Years)
C.
LAMP2A
| 100% |
| --- |
| 80% |
| 60% |
| 40% |
| 20% |
Proportion
| | 3+ |
| --- | --- |
| | 2+ |
| | 1+ |
p = 0.9248
| 0 | 1 | 2 | 3 | 4 | 5 |
| --- | --- | --- | --- | --- | --- |
Time (Years)
Supp. Fig. 6. Kaplan-Meier curves, showing overall survival of the St. Michael’s gastric cancer cohort, divided by A) cytoplasmic p62, B) LC3B and C) LAMP2A IHC staining patterns.
